# APPRAISE-AI Tool for Quantitative Evaluation of AI Studies for Clinical Decision Support

**DOI:** 10.1001/jamanetworkopen.2023.35377

**Published:** 2023-09-25

**Authors:** Jethro C. C. Kwong, Adree Khondker, Katherine Lajkosz, Matthew B. A. McDermott, Xavier Borrat Frigola, Melissa D. McCradden, Muhammad Mamdani, Girish S. Kulkarni, Alistair E. W. Johnson

**Affiliations:** 1 Division of Urology, Department of Surgery, University of Toronto, Toronto, Ontario, Canada; 2 Temerty Centre for AI Research and Education in Medicine, University of Toronto, Toronto, Ontario, Canada; 3 Department of Biostatistics, University Health Network, University of Toronto, Toronto, Ontario, Canada; 4 Department of Biomedical Informatics, Massachusetts Institute of Technology, Cambridge; 5 Laboratory for Computational Physiology, Harvard–Massachusetts Institute of Technology Division of Health Sciences and Technology, Cambridge; 6 Anesthesiology and Critical Care Department, Hospital Clinic de Barcelona, Barcelona, Spain; 7 Department of Bioethics, The Hospital for Sick Children, Toronto, Ontario, Canada; 8 Genetics & Genome Biology Research Program, Peter Gilgan Centre for Research and Learning, Toronto, Ontario, Canada; 9 Division of Clinical and Public Health, Dalla Lana School of Public Health, University of Toronto, Toronto, Ontario, Canada; 10 Data Science and Advanced Analytics, Unity Health Toronto, Toronto, Ontario, Canada; 11 Princess Margaret Cancer Centre, University Health Network, University of Toronto, Toronto, Ontario, Canada; 12 Division of Biostatistics, Dalla Lana School of Public Health, University of Toronto, Toronto, Ontario, Canada; 13 Child Health Evaluative Sciences, The Hospital for Sick Children, University of Toronto, Toronto, Ontario, Canada

## Abstract

**Importance:**

Artificial intelligence (AI) has gained considerable attention in health care, yet concerns have been raised around appropriate methods and fairness. Current AI reporting guidelines do not provide a means of quantifying overall quality of AI research, limiting their ability to compare models addressing the same clinical question.

**Objective:**

To develop a tool (APPRAISE-AI) to evaluate the methodological and reporting quality of AI prediction models for clinical decision support.

**Design, Setting, and Participants:**

This quality improvement study evaluated AI studies in the model development, silent, and clinical trial phases using the APPRAISE-AI tool, a quantitative method for evaluating quality of AI studies across 6 domains: clinical relevance, data quality, methodological conduct, robustness of results, reporting quality, and reproducibility. These domains included 24 items with a maximum overall score of 100 points. Points were assigned to each item, with higher points indicating stronger methodological or reporting quality. The tool was applied to a systematic review on machine learning to estimate sepsis that included articles published until September 13, 2019. Data analysis was performed from September to December 2022.

**Main Outcomes and Measures:**

The primary outcomes were interrater and intrarater reliability and the correlation between APPRAISE-AI scores and expert scores, 3-year citation rate, number of Quality Assessment of Diagnostic Accuracy Studies (QUADAS-2) low risk-of-bias domains, and overall adherence to the Transparent Reporting of a Multivariable Prediction Model for Individual Prognosis or Diagnosis (TRIPOD) statement.

**Results:**

A total of 28 studies were included. Overall APPRAISE-AI scores ranged from 33 (low quality) to 67 (high quality). Most studies were moderate quality. The 5 lowest scoring items included source of data, sample size calculation, bias assessment, error analysis, and transparency. Overall APPRAISE-AI scores were associated with expert scores (Spearman ρ, 0.82; 95% CI, 0.64-0.91; *P* < .001), 3-year citation rate (Spearman ρ, 0.69; 95% CI, 0.43-0.85; *P* < .001), number of QUADAS-2 low risk-of-bias domains (Spearman ρ, 0.56; 95% CI, 0.24-0.77; *P* = .002), and adherence to the TRIPOD statement (Spearman ρ, 0.87; 95% CI, 0.73-0.94; *P* < .001). Intraclass correlation coefficient ranges for interrater and intrarater reliability were 0.74 to 1.00 for individual items, 0.81 to 0.99 for individual domains, and 0.91 to 0.98 for overall scores.

**Conclusions and Relevance:**

In this quality improvement study, APPRAISE-AI demonstrated strong interrater and intrarater reliability and correlated well with several study quality measures. This tool may provide a quantitative approach for investigators, reviewers, editors, and funding organizations to compare the research quality across AI studies for clinical decision support.

## Key Points

**Question:**

Can quantitative methods be used to evaluate the robustness of artificial intelligence (AI) prediction models and their suitability for clinical decision support?

**Findings:**

In this quality improvement study, the APPRAISE-AI tool was developed to evaluate the methodological and reporting quality of 28 clinical AI studies using a quantitative approach. APPRAISE-AI demonstrated strong interrater and intrarater reliability and correlated well with other validated measures of study quality across a variety of AI studies.

**Meaning:**

These findings suggest that APPRAISE-AI fills a critical gap in the current landscape of AI reporting guidelines and provides a standardized, quantitative tool for evaluating the methodological rigor and clinical utility of AI models.

## Teaser

This quality improvement study evaluates the methodological and reporting quality of artificial intelligence (AI) models for clinical decision support.

## Introduction

Advances in computer science and data collection have fueled the development of artificial intelligence (AI) applications across the health care sector in recent years. This proliferation of AI in medicine has been met with major interest from various stakeholders, including patients, practitioners, and even regulatory bodies such as the US Food and Drug Administration. Although much of the initial excitement for these novel AI solutions has been centered around their performance, there has been growing attention toward ensuring the reproducibility, safety, and fairness of these applications.^1^ Indeed, recent work^2^ has highlighted several methodological concerns within the existing clinical AI literature, including poor adherence to conventional reporting guidelines, inadequate sample size (ie, low number of events per variable), no external validation, limited assessment of calibration, and bias.

These concerns have prompted the development of several reporting guidelines along the AI pathway, including MI-CLAIM, TRIPOD-AI, and STARD-AI for model development^3,4,5^; DECIDE-AI for model evaluation^6^; and CONSORT-AI and SPIRIT-AI for clinical trials evaluation.^7,8^ Other reporting guidelines have also been adopted within various clinical domains, including cardiology (PRIME),^9^ dentistry,^10^ medical imaging (Radiomics Quality Score),^11^ ophthalmology,^12^ and urology (STREAM-URO).^13^ These guidelines are valuable in ensuring transparency, reproducibility, and comparability in AI research by providing a list of minimum reporting items for AI studies. However, they nevertheless do not provide a means of quantifying the overall quality of clinical AI research, which necessitates evaluating methodological soundness, appropriateness to clinical targets, and more. This lack of a quantitative assessment tool makes it difficult to evaluate the robustness of AI models and their readiness for clinical use, particularly when comparing 2 models addressing the same clinical question.

Given this substantial gap, there is a pressing need for a validated tool that not only assesses the methodological and reporting quality of AI studies in health care but also provides a standardized, quantitative measure of their clinical utility and safety. Such a tool would be of immense value to investigators, reviewers, and funding organizations, enabling them to compare the quality of research across AI studies and facilitate safer and more effective integration of AI tools into clinical practice.

Here, we propose the APPRAISE-AI tool, an instrument to evaluate the methodological and reporting quality of AI studies for clinical decision support. We demonstrate its validity and reliability on existing AI literature. Finally, we provide examples on how to use APPRAISE-AI to assess the most common types of clinical AI studies, including image analysis, survival analysis, and classification.

## Methods

### Development of APPRAISE-AI

 Ethics approval and informed consent were not needed for this quality improvement study because it involved a systematic review of published studies and did not involve patient data, in accordance with 45 CFR §46. This project was conducted in compliance with the Standards for Quality Improvement Reporting Excellence (SQUIRE) reporting guideline.^14^

APPRAISE-AI was designed to evaluate primary studies that develop, validate, or update any machine learning model for clinical decision support. Candidate items were initially generated following a literature review of existing reporting guidelines on AI in medicine.^13^ These items were further refined through critical discussion by a panel of experts in clinical AI research, which included clinicians (J.C.C.K., A.K., X.B.F., and G.S.K.), AI experts (M.B.A.M., X.B.F., M.M., and A.E.W.J.), clinical epidemiologists (K.L. and G.S.K.), bioethicists (M.D.M.), and journal editors (A.E.W.J.). Item descriptions were modified from our previous reporting guideline.^13^ Scores were then assigned to each APPRAISE-AI item, with higher scores reflecting stronger methodological or reporting quality.

The final APPRAISE-AI tool consisted of 24 items with a maximum overall score of 100 points (eTable 1 in Supplement 1). Points were weighted more heavily toward methods (items 4-12, of 51 points), results (items 13-19, of 27 points), and transparency (item 24, of 10 points), because these areas were commonly underreported according to previous reviews.^2^ Scoring options for each item were assigned on the basis of current best practices in AI and prediction model reporting. For example, items 1 to 3, 5, 11 to 14, and 20 to 23 were scored according to recommendations from the Transparent Reporting of a Multivariable Prediction Model for Individual Prognosis or Diagnosis (TRIPOD) reporting guideline,^15^ a well-established reporting guideline for prediction models in the medical literature.

For model development, generalizability of supervised learning models is best achieved through training on diverse, representative data with annotated labels that accurately reflect the clinical problem (items 4 and 6, respectively).^16^ APPRAISE-AI assesses data sources on the basis of routinely captured proxies of patient diversity, including number of institutions, health care setting, and geographical location. Although model evaluation in multiple countries represents a high bar of evidence, it is deemphasized in APPRAISE-AI compared with other measures because of its inherent logistical complexity. Instead, a greater focus is placed on incorporating historically underrepresented groups, such as community-based, rural, or lower income populations. Data preprocessing steps (item 7) are recognized as important components in both non-AI and AI reporting guidelines including how data were abstracted, how missing data were handled, and how features were modified, transformed, and/or removed.^6,15^ Methods to address class imbalance were excluded because recent simulation studies have shown that imbalance correction may worsen model calibration despite no clear improvement in discrimination.^17^ Data splitting (item 8) was graded according to established hierarchies of validation strategies.^18^ Although there is no universally accepted method for determining minimum sample size for AI model development and validation,^19^ prior simulation studies have shown that AI models may require “at least 10 events per variable”^20^ to achieve stable performance (item 9).

For model evaluation, item 10 reflects the importance of comparing AI models against the accepted reference standard (eg, clinician judgment), regression approaches, and/or existing models.^21^ Although area under the receiver operating characteristic curve is commonly reported to characterize model performance, other measures may be more relevant, depending on the clinical context (item 15). For example, researchers may wish to consult the Metrics Reloaded recommendations for image analysis.^22^ Other measures that assess model calibration, or the level of agreement between predictions and observed outcomes, should be considered. In particular, quantifying net benefit through decision curve analysis enables one to determine whether their AI model is doing more good than harm (item 16).^23^ Ratings used to assess quality of bias assessment are based on patient-specific or task-specific subgroup analysis and exploratory error analysis from the medical algorithmic audit proposed by Liu and colleagues (items 17 and 18, respectively).^1^ Model explanations (item 19) are considered optional at this time because of limitations with consistency and reliability.^24^ Finally, item 24 emphasizes the importance of addressing the ongoing reproducibility crisis in AI research, by promoting the practice of making research data and models publicly available to enable the replication and verification of findings.^25^

Each APPRAISE-AI item was mapped to one of the following domains: clinical relevance, data quality, methodological conduct, robustness of results, reporting quality, and reproducibility (Table 1). Scores could then be tabulated to determine the overall study quality (overall APPRAISE-AI score) and domain quality (APPRAISE-AI domain score).

**Table 1.** APPRAISE-AI Domains and Corresponding Items^a^

Domain and items	Domain score
Clinical relevance	
1. Title	4
2. Background	
3. Objective and problem	
21. Implementation into clinical practice	
Data quality	
4. Source of data	24
5. Eligibility criteria	
6. Ground truth	
7. Data abstraction, cleaning, preparation	
Methodological conduct	
8. Data splitting	20
9. Sample size calculation	
10. Baseline	
Robustness of results	
15. Model evaluation	20
16. Clinical utility assessment	
17. Bias assessment	
18. Error analysis	
19. Model explanation	
Reporting quality	
13. Cohort characteristics	12
20. Critical analysis	
22. Limitations	
23. Disclosures	
Reproducibility	
11. Model and processing description	20
12. Hyperparameter tuning	
14. Model specification	
24. Transparency	
Overall score	100

^a^ Please refer to eTable 1 in Supplement 1 for a detailed breakdown of each item. The overall APPRAISE-AI score was graded as follows: very low quality, 0 to 19; low quality, 20 to 39; moderate quality, 40 to 59; high quality, 60 to 79; and very high quality, 80 to 100.

### Using APPRAISE-AI to Assess AI Studies to Predict Sepsis

The APPRAISE-AI tool was applied to a recent systematic review on machine learning to predict sepsis, which included articles published until September 13, 2019.^26^ Each article was independently graded by 2 raters using the APPRAISE-AI tool. For items that indicate “select one of the following,” raters were instructed to score the highest possible value where applicable. For example, if a study provided both internal (+1) and external (+3) validation, a score of 3 was recorded for item 8. All other items were considered “select all that apply.” For example, if a study included data from multiple countries (+1) and community hospitals (+2), a score of 3 would be assigned for item 4.

Three experts (A.E.W.J., M.B.A.M., and X.B.F.) in clinical AI research independently assessed each article according to 8 criteria using a scale of 1 to 5 (1, very weak; 5, very strong) (eTable 2 in Supplement 1). Criteria scores were summed to generate an overall expert score for each article (maximum overall score of 40 points). All information regarding the authors, affiliations, institutions, source of funding, and journal for each article were redacted to mask both groups. Assessors did not have access to other assessors’ scores. We provide additional detailed examples and explanations of high-quality studies for various study types, including image analysis, classification, and survival analysis in eTables 3, 4, and 5 in Supplement 1.

### Statistical Analysis

#### Validity of APPRAISE-AI

Spearman ρ was used to assess construct validity in the following ways. First, the correlation between median overall APPRAISE-AI and expert scores was measured. Second, the association of overall APPRAISE-AI scores with 3-year citation rate, defined as the number of nonself citations from the Scopus database within the first 3 years of publication, was measured. This time frame was selected because all articles were published at least 3 years before this study. Finally, APPRAISE-AI was compared against other widely used tools, including the Quality Assessment of Diagnostic Accuracy Studies (QUADAS-2) criteria and the TRIPOD statement.^15,27^ Specifically, the associations of overall APPRAISE-AI scores with number of QUADAS-2 low risk-of-bias domains and overall adherence to TRIPOD were measured.

#### Reliability of APPRAISE-AI

Intraclass correlation coefficients (ICCs; calculated with 2-way random effects, absolute agreement, and single measurement) were used to measure interrater and intrarater reliability for each APPRAISE-AI item and domain. For intrarater reliability, each article was regraded by the same nonexpert raters (J.C.C.K. and A.K.) 3 months after the first assessment. ICC interpretation was based on Koo et al,^28^ in which ICC values less than 0.50 indicated poor reliability, values of 0.50 to 0.75 indicated moderate reliability, values of 0.75 to 0.90 indicated good reliability, and values greater than 0.90 indicated excellent reliability.

#### Sample Size Calculations

A sample size of 28 studies was sufficient to achieve at least 80% power to detect a Spearman ρ of 0.53 or higher and an ICC of 0.45 or higher, assuming a significance level of 2-sided *P* < .05 (eAppendix in Supplement 1). All analyses were conducted using SPSS version 26 (IBM). Data analysis was performed from September to December 2022.

## Results

### Quality of AI Studies to Predict Sepsis

A total of 28 studies were included, published between 2010 and 2019. Of these, 24 described AI models in the model development phase. One study^29^ included both silent trial and single-group clinical trial phases. Two studies^30,31^ evaluated their AI models through single-group clinical trials, whereas another study^32^ conducted a randomized clinical trial comparing their AI model against the standard of care. The APPRAISE-AI scores are summarized in the Figure. The median overall score was 48 (moderate quality) and ranged from 33 (low quality) to 67 (high quality), with 22 of 28 studies considered moderate quality (see the Data Sharing Statement in Supplement 2). The overall quality of studies did not improve over time from 2010 to 2019 (correlation coefficient, 0.12; 95% CI, −0.26 to 0.48; *P* = .53). All studies that prospectively evaluated their models, either through silent or clinical trial phases, achieved at least moderate overall quality. The 5 lowest scoring items, based on percentage of the maximum possible score for each item, were source of data, sample size calculation, bias assessment, error analysis, and transparency. Although studies performed well in the clinical relevance and reporting quality domains, they had lower scores in methodological conduct, robustness of results, and reproducibility.

**Figure.**
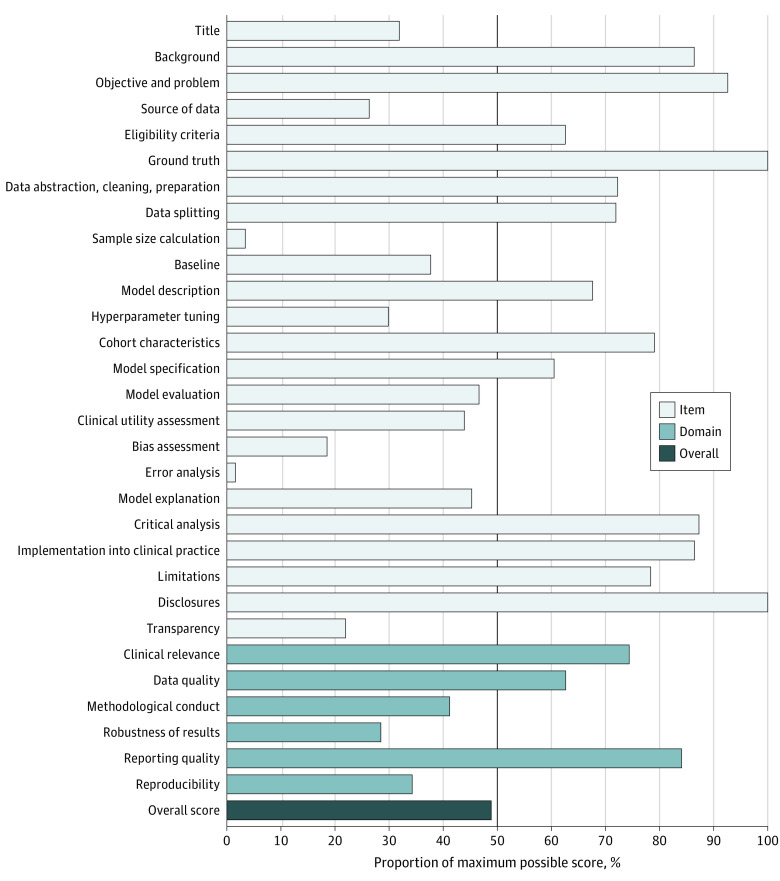
Mean APPRAISE-AI Item, Domain, and Overall Scores for the 28 Studies Using Artificial Intelligence to Predict Sepsis Each field is presented as a percentage of the maximum possible score for that field (ie, mean score / maximum possible score × 100%) to compare scores between fields, irrespective of the assigned weighting.

### Validity and Reliability of APPRAISE-AI

Overall APPRAISE-AI scores were highly correlated with consensus expert ratings (Spearman ρ, 0.82; 95% CI, 0.64-0.91; *P* < .001) (Table 2). In addition, overall APPRAISE-AI scores were significantly associated with 3-year citation rates (Spearman ρ, 0.69; 95% CI, 0.43-0.85; *P* < .001), number of low risk-of-bias domains on QUADAS-2 (Spearman ρ, 0.56; 95% CI, 0.24-0.77; *P* = .002), and overall adherence to TRIPOD (Spearman ρ, 0.87; 95% CI, 0.73-0.94; *P* < .001).

**Table 2.** Association of Overall APPRAISE-AI Scores With Other Measures of Study Quality

Measure	Spearman ρ (95% CI)	*P* value
Consensus expert score	0.82 (0.64-0.91)	<.001
3-y Citation rate	0.69 (0.43-0.85)	<.001
No. of low risk-of-bias domains on Quality Assessment of Diagnostic Accuracy Studies–2	0.56 (0.24-0.77)	.002
Adherence to Transparent Reporting of a Multivariable Prediction Model for Individual Prognosis or Diagnosis reporting guideline	0.87 (0.73-0.94)	<.001

Interrater reliability was moderate to excellent, with ICCs ranging from 0.74 to 1.00 for item scores, 0.81 to 0.92 for domain scores, and 0.91 for overall scores (Table 3). APPRAISE-AI also demonstrated moderate to excellent intrarater reliability, with ICCs ranging from 0.74 to 1.00 for item scores, 0.89 to 0.99 for domain scores, and 0.98 for overall scores.

**Table 3.** Interrater and Intrarater Reliability of APPRAISE-AI Items, Domains, and Overall Score Determined by ICCs

Variable	ICC (95% CI)^a^	
	Interrater reliability	Intrarater reliability
Item		
Title	0.76 (0.61-0.86)	0.76 (0.62-0.85)
Background	0.77 (0.64-0.86)	0.77 (0.64-0.86)
Objective and problem	0.74 (0.59-0.84)	0.74 (0.59-0.84)
Source of data	0.90 (0.80-0.95)	0.99 (0.98-0.99)
Eligibility criteria	0.77 (0.54-0.87)	0.90 (0.84-0.94)
Ground truth	1.00	1.00
Data abstraction, cleaning, preparation	0.80 (0.67-0.88)	0.98 (0.97-0.99)
Data splitting	0.75 (0.61-0.84)	1.00
Sample size calculation	1.00	1.00
Baseline	0.83 (0.72-0.89)	0.97 (0.95-0.98)
Model description	0.77 (0.63-0.86)	0.94 (0.90-0.97)
Hyperparameter tuning	0.76 (0.62-0.85)	0.96 (0.92-0.98)
Cohort characteristics	0.80 (0.68-0.88)	0.98 (0.96-0.99)
Model specification	0.81 (0.69-0.88)	0.90 (0.84-0.94)
Model evaluation	0.79 (0.66-0.87)	0.96 (0.94-0.98)
Clinical utility assessment	0.78 (0.63-0.87)	0.95 (0.91-0.97)
Bias assessment	0.79 (0.62-0.89)	0.96 (0.94-0.98)
Error analysis	1.00	1.00
Model explanation	0.82 (0.71-0.89)	0.96 (0.94-0.98)
Critical analysis	0.84 (0.74-0.90)	1.00
Implementation into clinical practice	0.77 (0.64-0.86)	0.92 (0.87-0.95)
Limitations	0.79 (0.67-0.87)	1.00
Disclosures	1.00	1.00
Transparency	0.95 (0.92-0.97)	0.99 (0.99-1.00)
Domain		
Clinical relevance	0.83 (0.70-0.90)	0.89 (0.80-0.94)
Data quality	0.82 (0.70-0.89)	0.97 (0.95-0.98)
Methodological conduct	0.85 (0.75-0.91)	0.98 (0.97-0.99)
Robustness of results	0.81 (0.63-0.90)	0.94 (0.90-0.96)
Reporting quality	0.86 (0.78-0.92)	0.99 (0.99-1.00)
Reproducibility	0.92 (0.86-0.95)	0.99 (0.98-1.00)
Overall score	0.91 (0.85-0.95)	0.98 (0.96-0.99)

Abbreviation: ICC, intraclass correlation coefficient.

^a^ ICCs were calculated with 2-way random effects, absolute agreement, and single measurement.

## Discussion

There is growing recognition toward ensuring a safe and ethical implementation of AI tools into clinical practice. However, recent evidence suggests that many AI studies fail to follow best practices in developing prediction models.^2,25^ There remains a need for a standardized tool to quantify the robustness and clinical utility of AI models. In this quality improvement study, the APPRAISE-AI tool addresses this gap and differs from current AI reporting checklists by providing additional granularity in the assessment of methodological and reporting quality. Each APPRAISE-AI item assigns different point values on the basis of prespecified criteria that reflect current best practices in AI. By providing an overall and domain-specific score (clinical relevance, data quality, methodological conduct, robustness of results, reporting quality, and reproducibility), APPRAISE-AI enables researchers to gain both macro-level and micro-level insights on the quality of evidence generated to support their AI models.

A recent systematic review^26^ revealed a high risk of bias among the majority of studies (68%) included in this analysis. These studies covered various phases of the AI life cycle, from model development to clinical trial assessment. APPRAISE-AI was applicable in all settings and demonstrated moderate to excellent interrater and intrarater reliability. Furthermore, it correlated well with other validated measures of study quality, including expert ratings, QUADAS-2, TRIPOD, and 3-year citation rates. APPRAISE-AI highlighted additional AI-specific limitations of each study. The 3 lowest domains identified were methodological conduct, robustness of results, and reproducibility, which arguably are the most important characteristics in determining the scientific rigor and generalizability of an AI model. Overall study quality ranged from low to high, with the majority of studies demonstrating moderate quality.

APPRAISE-AI offers a standardized framework for the quantitative evaluation of AI studies for clinical decision support, which may be a useful resource for conducting systematic reviews. To illustrate its utility, we provide detailed examples and explanations of high-quality studies for various study types, including image analysis, classification, and survival analysis (eTables 3, 4, and 5 in Supplement 1). Other potential applications include use by funding agencies to inform grant allocation for AI research, by journal editors to prescreen submitted articles, and by implementers to survey the field for high-quality AI tools. For instance, hospitals may wish to consider only AI models that are deemed high or very high quality according to APPRAISE-AI because they have the highest scientific rigor and the greatest potential in improving patient outcomes.

### Limitations

Several limitations merit discussion. Although the construct validity of the APPRAISE-AI tool was successfully demonstrated using a previously published systematic review of sepsis AI models,^26^ a considerable proportion of those studies (36%) used the Medical Information Mart for Intensive Care database. As such, the variability of data quality domain scores may have been limited. Therefore, use of the APPRAISE-AI tool in larger systematic reviews with more diverse data sets may yield a wider range of quality. Second, study citation rates may not be a reliable measure of quality; however, we attempted to mitigate this limitation by excluding self-citations. Furthermore, APPRAISE-AI was well-correlated with other validated measures of study quality, such as QUADAS-2 and TRIPOD. Third, this iteration of APPRAISE-AI is based on current best practices in AI. However, as AI methods continue to evolve at a rapid pace, this tool may need to be updated to reflect these advancements. For example, model explainability remains a highly controversial topic among clinical and AI experts, with no universally accepted method for providing robust explanations for individual-level predictions.^33^ Similarly, there is no clear consensus on the best strategy to incorporate algorithmic fairness considerations^34,35^; therefore, APPRAISE-AI does not assign scores to any particular approach. Instead, the emphasis is placed on conducting bias assessments (item 17) so that researchers can examine the efficacy of their fairness strategies, regardless of the approach used.

It must be emphasized that APPRAISE-AI, like other reporting guidelines, cannot replace clinical and methodological expertise. For example, even if a study uses an objective, well-captured ground truth (ie, the highest assigned score for item 6, quality of ground truth), it may not be appropriate for the specific clinical problem. In addition, even the performance of a very high quality AI model may degrade over time or when applied to a foreign setting owing to data set and concept drift.^36,37^ This issue has been exemplified by the Epic Sepsis Model, which substantially underperformed on external validation.^38^ Another consideration is that APPRAISE-AI is not intended to evaluate feasibility and other ethical considerations that are essential to clinical implementation, such as ease of use, interoperability, and privacy concerns. Furthermore, APPRAISE-AI is primarily intended for AI research focused on clinical decision support and may be less applicable for other types of studies, such as causal inference.

## Conclusions

APPRAISE-AI has a broad range of applications for clinicians, researchers, scientific journals, funding organizations, and regulatory bodies to assess the methodological and reporting quality of clinical AI research. APPRAISE-AI may further enhance investigator transparency and accountability during the model development and validation phases. We hope that this tool will empower researchers to generate higher quality evidence to support their AI studies. We invite the AI community to provide feedback and suggestions on this iteration of the APPRAISE-AI tool, which is available in a public repository.

## Notes

Supplement 1. **eAppendix.** Supplemental Methods **eTable 1.** The APPRAISE-AI Tool to Assess Quality of AI Studies in Medicine **eTable 2.** Scoring Rubric for Expert Ratings for Each Included Article **eTable 3.** APPRAISE-AI Tool on a High-Quality Image Analysis Study **eTable 4.** APPRAISE-AI Tool on a High-Quality Classification Study **eTable 5.** APPRAISE-AI Tool on a High-Quality Survival Analysis Study

Supplement 2.
Data Sharing Statement
