# Supplementary material for: APPRAISE-AI Tool for Quantitative Evaluation of AI Studies for Clinical Decision Support
Source: JAMA Netw Open. 2023 Sep 25;6(9):e2335377. doi: 10.1001/jamanetworkopen.2023.35377 (PMC10520738; doi:10.1001/jamanetworkopen.2023.35377)
Supplement: Supplement 2. — Data Sharing Statement [file jamanetwopen-e2335377-s002.pdf]

Data Sharing Statement

Kwong. APPRAISE-AI Tool for Quantitative Evaluation of AI Studies for Clinical Decision Support. *JAMA Netw Open*. Published September 25, 2023.
doi:10.1001/jamanetworkopen.2023.35377

Data
Data available: Yes
Data types: Data (not involving human participants)
How to access data: https://doi.org/10.5281/zenodo.7930888
When available: With publication

Supporting Documents
Document types: None

Additional Information
Who can access the data: Publicly available
Types of analyses: For any purpose
Mechanisms of data availability: Publicly available
